# Berberine nanoparticles protects tubular epithelial cells from renal ischemia-reperfusion injury

**DOI:** 10.18632/oncotarget.16530

**Published:** 2017-03-23

**Authors:** Da Xie, Yong Xu, Wang Jing, Zeng Juxiang, Li Hailun, Hu Yu, Dong-Hui Zheng, Yong-Tao Lin

**Affiliations:** ^1^ Department of Nephrology, The Second Affiliated Hospital of Nanjing Medical University, Nanjing, China; ^2^ Department of Nephrology, Huaian Hospital Affiliated to Xuzhou Medical University and Huaian Second Hospital, Huaian, China; ^3^ Department of Pediatrics, Huaian Hospital Affiliated to Xuzhou Medical University and Huaian Second Hospital, Huaian, China; ^4^ Jiangsu College of Nursing, Huaian, Jiangsu, China

**Keywords:** renal ischemia-reperfusion, berberine, nanoparticles, oxidative stress, apoptosis, Pathology Section

## Abstract

Renal ischemia-reperfusion (I/R) injury is one of the most common causes of acute renal failure, the prognosis of which remains poor and there still lacks of effective therapeutics available in the clinic. This study aimed at investigating the effects of Berberine nanoparticles (BBR-NP) on the ischemia-reperfusion injury of renal tubular epithelial cells and underlying the mechanisms. Our results showed that in a rat model of renal I/R injury, BBR and BBR-NP protected renal against injury both functionally (as assessed by serum urea nitrogen and creatinine level) and morphologically (as assessed by HE staining, transmission electron microscopy and TUNEL staining) in a dose-dependent manner, with the effects of BBR-NP superior to BBR alone. Mechanism investigation showed that BBR-NP reversed oxidative stress and subsequent apoptosis of renal cells, as demonstrated by the decreased expression of proteins involved in the oxidative stress and mitochondrial stress pathways. In conclusion, our study showed that BBR-NP is superior to BBR alone in protecting renal against I/R injury and explored the underlying mechanisms, which should be tested in further studies and might give impetus to the development of novel therapeutics based on BBR-NP against renal I/R.

## INTRODUCTION

Renal ischemia-reperfusion (I/R) injury is one of the most common causes of acute renal failure after renal transplantation, shock, sepsis, and renal artery stenosis [1], which results in high rates of morbidity and mortality. Currently, the prognosis for patients with I/R injury is poor and there is no effective therapy available to treat this injury. Although the exact mechanisms of I/R injury remain undefined, studies have showed that excessive amount of ROS produced by the damaged tissue can cause oxidative stress which changes mitochondrial oxidative phosphorylation, ATP depletion, increase intracellular calcium and activation of membrane phospholipids proteases [2–4]. Oxygen free radicals produced during I/R injury may lead to renal injury by lipid peroxidation [5]. Therefore, antioxidants has the potentional to decrease the I/R injury.

Berberine (BBR) is an isoquinoline alkaloid extract from traditional medicine herbs. It has been used to treat diarrhea in oriental medicine since long time ago [6]. In the last few decades many clinical studies have well established the antioxidant actions of berberine in various disorders ranging from diabetes [7], high cholesterol [8], various inflammatory conditions [8] and CNS disorders [9] such as Alzheimer, cerebral ischemia. Other study about neonatal rat cardiomyocytes showed that BBR could reduce norepinephrine-induced apoptosis through inhibiting the ROS-TNF-a-caspase signaling pathway [10]. Additionally, another study showed that BBR demonstrated compelling renoprotective effect in renal I/R injury [11]. However, the clinical application of BBR is greatly limited due to poor gastrointestinal absorption and thereafter low plasma levels and poor bioavailability after oral administration. Besides, high doses (0.9-1.5g/day) of BBR usually leads to gastrointestinal side effects as a result of its poor absorption and long-term administration [12]. Therefore, novel dosage forms of BBR that can improve its absorption and bioavailability is needed. Nanoparticles(NP) have been used to localize BBR to the gastric epithelium for the treatment of H. pylori infection [13]. It has also been reported that berberine nanoparticles(BBR-NP) can ameliorate hepatosteatosis in db/db mice [14]. It is therefore assumed that NP can enhance the absorption and bioavailability of BBR for the treatment of renal I/R injury. Therefore, the present study investigated whether BBR-NP was able to prevent renal I/R injury more efficiently in a rat model and to determine the mechanism in that process.

## RESULTS

### Functional protection of renal I/R injury by BBR-NP

**Figure 1:**
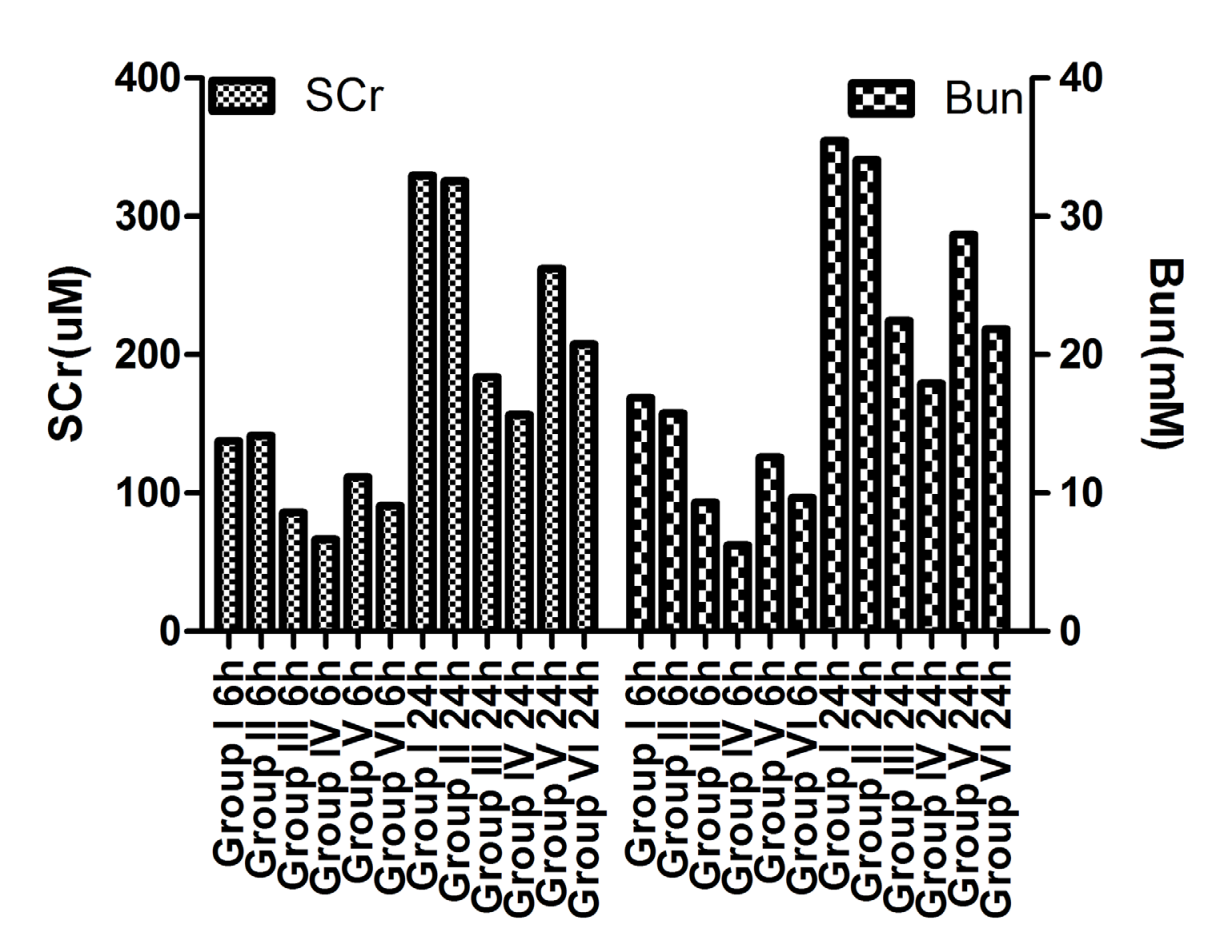
Serum urea nitrogen levels and creatinine levels at 6h or 24h after renal I/R injury in different groups Group I: control **A**. (saline with 10% DMSO), Group II: control **B**. (NP), Group III: Low dose BBR-NP (2mg/kg), Group IV: High dose BBRNP(4mg/kg), Group V: Low dose BBR(2mg/kg), Group VI: High dose BBR (4mg/kg).

Renal function was assessed by measuring serum urea nitrogen and creatinine in a clinical laboratory. BBR caused dramatic decrease in both serum urea nitrogen levels and creatinine levels compared with Control A and Control B groups. BBR-NP caused even higher decrease in in both serum urea nitrogen levels and creatinine levels compared with BBR groups. Higher dose of BBR or BBR-NP caused dramatic decrease in both serum urea nitrogen levels and creatinine levels compared with corresponding lower dose group (Figure 1). The same goes for both 6 hours and 24 hours after renal I/R injury.

### Renal histological evaluation

**Figure 2:**
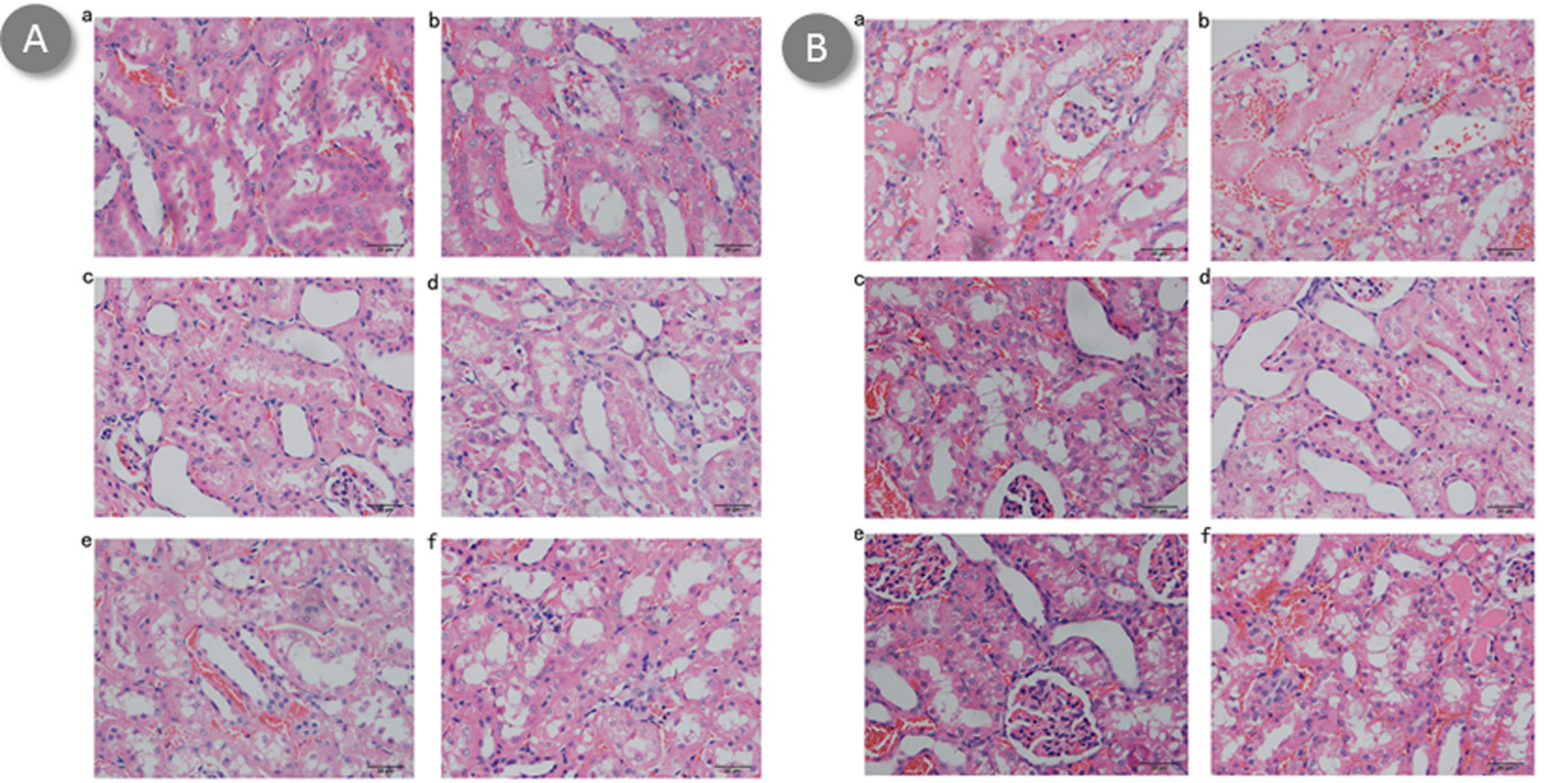
Effect of BBR-NP on renal I/R injury-induced alteration using HE staining at 6h (A) and 24 h (B) **A**. (a) control A: (saline with 10% DMSO), (b) Group II: control B: (NP), (c) Group III: Low dose BBR-NP (2mg/kg), (d) Group IV: High dose BBRNP(4mg/kg), (e) Group V: Low dose BBR(2mg/kg), (f) Group VI: High dose BBR(4mg/kg). **B**. (a) control A: (saline with 10% DMSO), (b) Group II: control B: (NP), (c) Group III: Low dose BBR-NP (2mg/kg), (d) Group IV: High dose BBRNP(4mg/kg), (e) Group V: Low dose BBR(2mg/kg), (f) Group VI: High dose BBR(4mg/kg).

**Figure 3:**
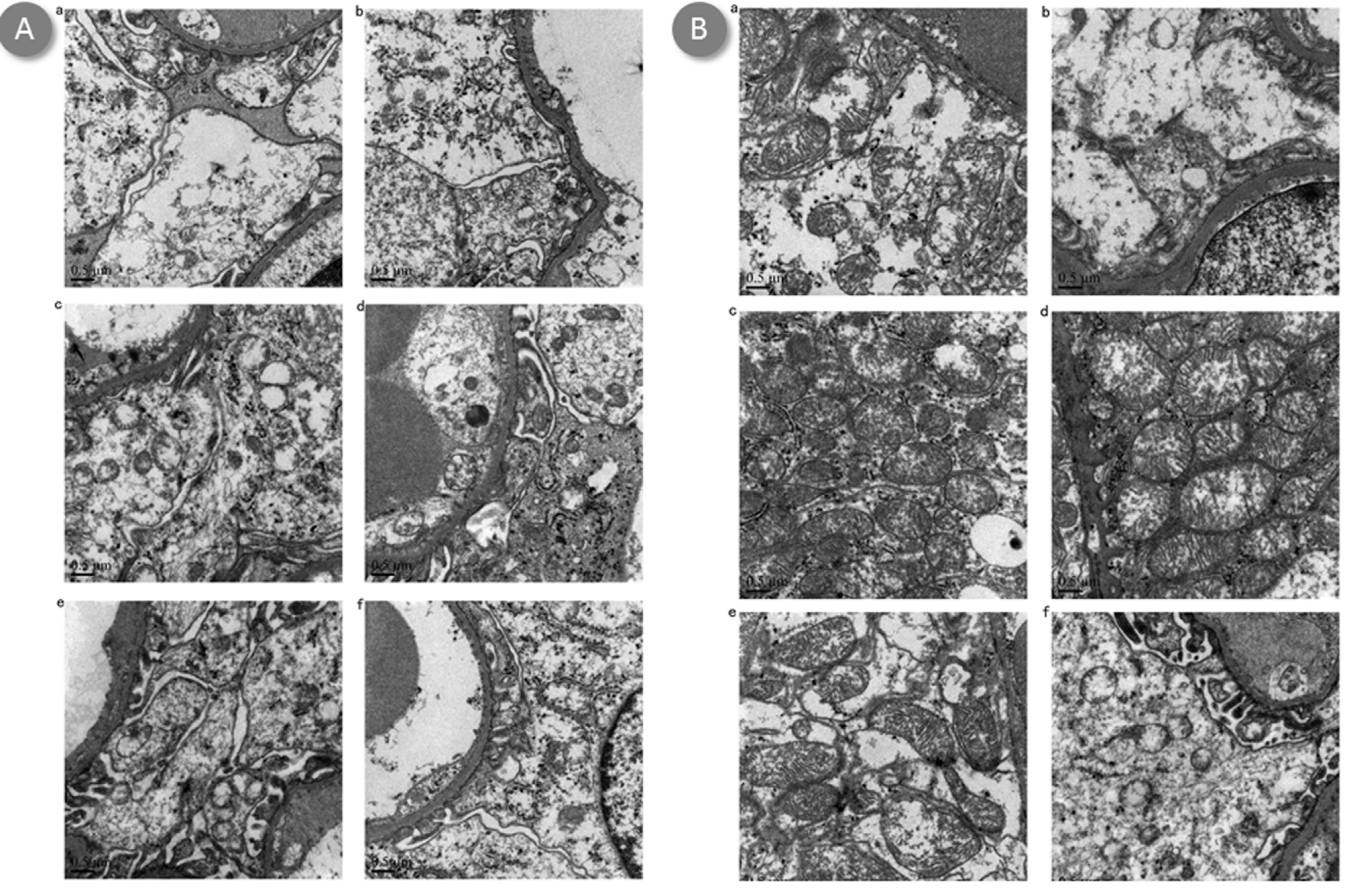
Effect of BBR-NP on renal I/R injury -induced alteration using 0.5um transmission electron microscopy at 6h (A) and 24h (B) **A**. (a) control A: (saline with 10% DMSO), (b) Group II: control B: (NP), (c) Group III: Low dose BBR-NP (2mg/kg), (d) Group IV: High dose BBRNP(4mg/kg), (e) Group V: Low dose BBR(2mg/kg), (f) Group VI: High dose BBR(4mg/kg). **B**. (a) control A: (saline with 10% DMSO), (b) Group II: control B: (NP), (c) Group III: Low dose BBR-NP (2mg/kg), (d) Group IV: High dose BBRNP(4mg/kg), (e) Group V: Low dose BBR(2mg/kg), (f) Group VI: High dose BBR(4mg/kg).

**Figure 4:**
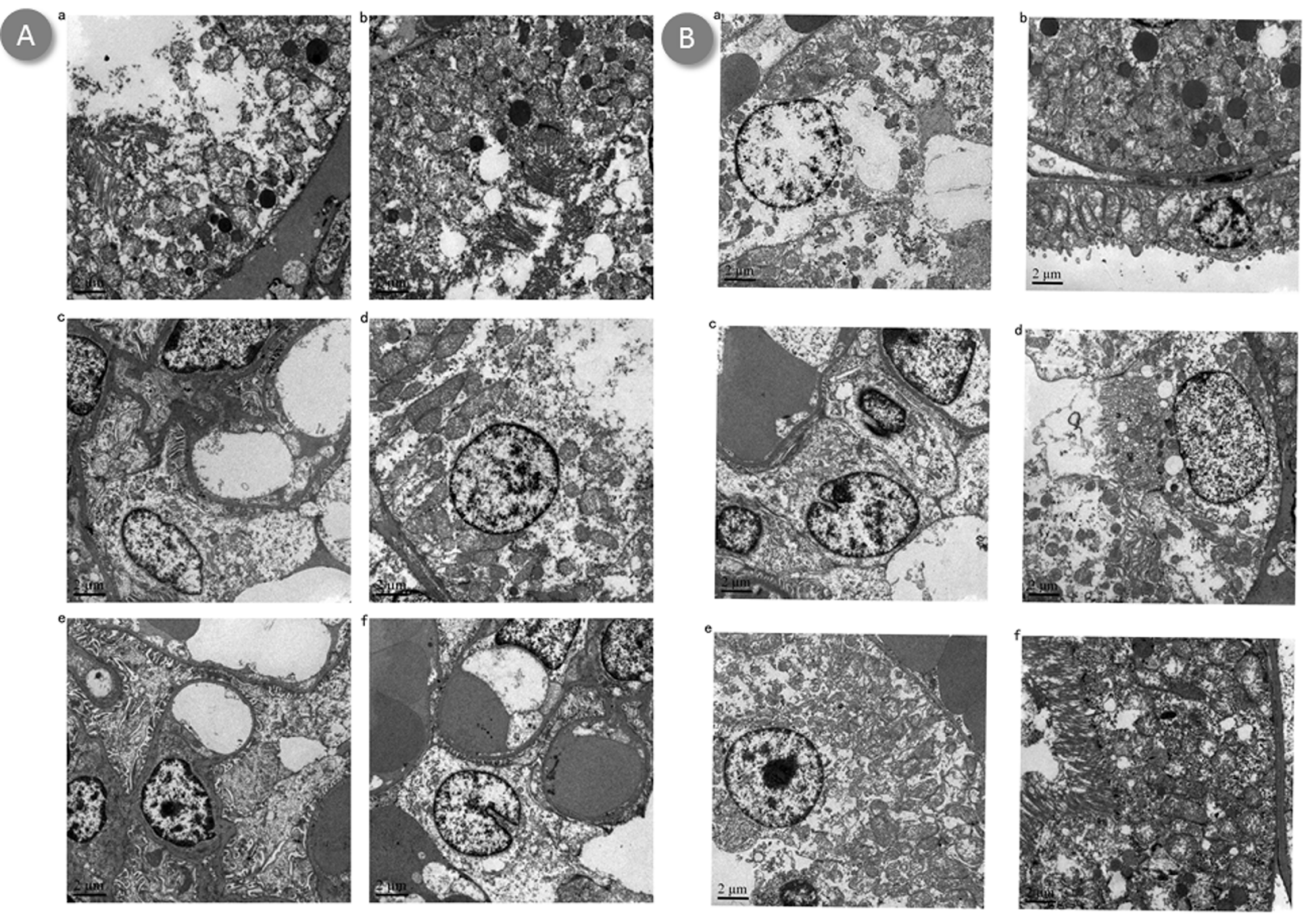
Effect of BBR-NP on renal I/R injury -induced alteration using 2.0 um transmission electron microscopy at 6h (A) and 24h (B) **A**. (a) control A: (saline with 10% DMSO), (b) Group II: control B: (NP), (c) Group III: Low dose BBR-NP (2mg/kg), (d) Group IV: High dose BBRNP(4mg/kg), (e) Group V: Low dose BBR(2mg/kg), (f) Group VI: High dose BBR(4mg/kg). **B**. (a) control A: (saline with 10% DMSO), (b) Group II: control B: (NP), (c) Group III: Low dose BBR-NP (2mg/kg), (d) Group IV: High dose BBRNP(4mg/kg), (e) Group V: Low dose BBR(2mg/kg), (f) Group VI: High dose BBR(4mg/kg).

Untreated mice demonstrated increased neutrophil infiltrations and more tubular necrosis than BBR or BBR-NP-treated mice in HE staining, BBR-treated mice demonstrated increased neutrophil infiltration and more tubular necrosis than BBR-NP-treated mice in HE staining at 6-hour (Figure 2A) or 24-hour (Figure 2B) post renal I/R injury. Under transmission electron microscopy, the effacement of podocyte foot processes, mitochondria vacuoles and cell fragments can be seen in the Control A and Control B group (Figures 3 and 4). Renal tubular damage, glomerular damage, mitochondrial damage and necrotic area can be seen in the 24h hour group (Figure 3B, Figure 4B). These damages wereless in the low dose BBR group than those in the aforementioned two control groups. It is obvious to see swelling in the glomerular capillary, renal tubular damage, fragmented epithelial cells and mitochondria damage (Figures 3 and 4). Although changes in the glomerular capillary, renal tubular damage, fragmented epithelial cells and mitochondria can still be observed in the high-dose group, they were less compared with those in the low-dose BBR group, with (Figures 3 and 4).

### Renal apoptosis measurement

**Figure 5:**
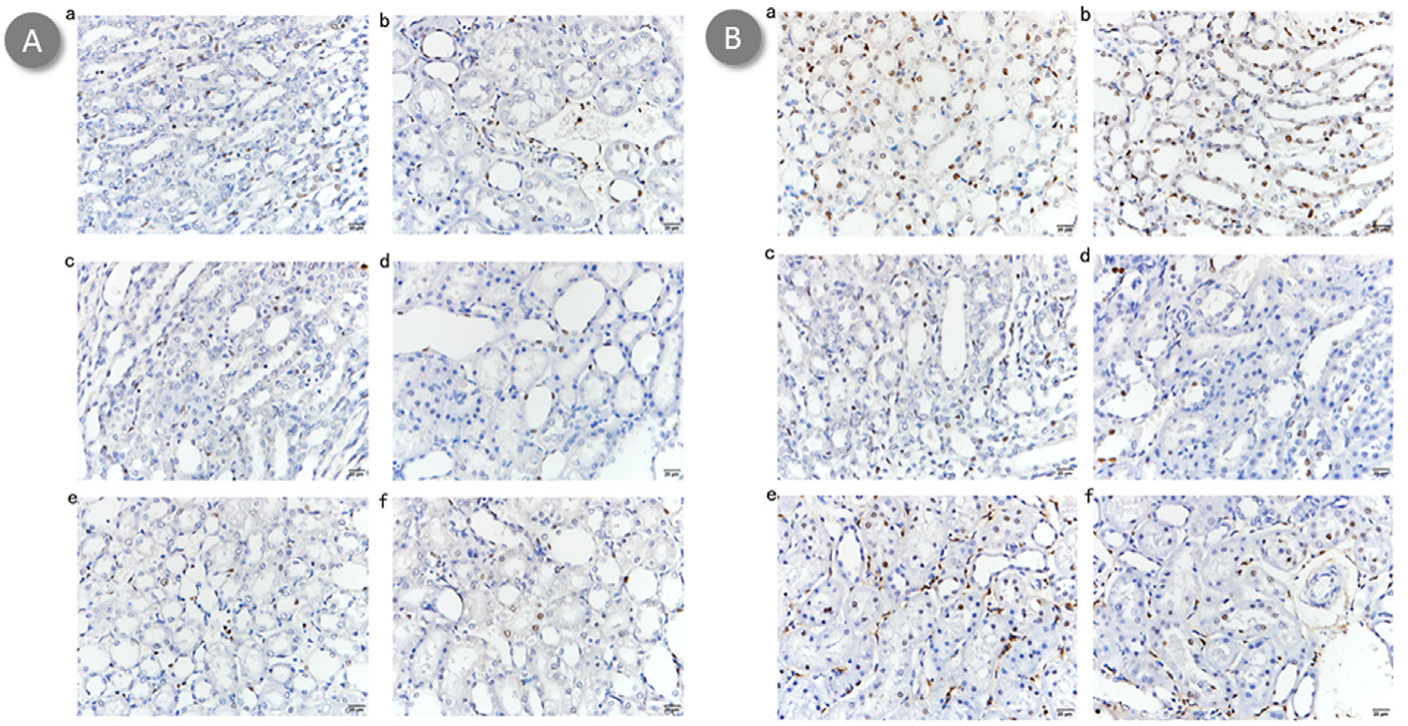
Effect of BBR-NP on renal I/R injury-induced apoptosis TUNEL staining at 6h using (A) and 24h (B) **A**. (a) control A: (saline with 10% DMSO), (b) Group II: control B: (NP), (c) Group III: Low dose BBR-NP (2mg/kg), (d) Group IV: High dose BBRNP(4mg/kg), (e) Group V: Low dose BBR(2mg/kg), (f) Group VI: High dose BBR(4mg/kg). **B**. (a) control A: (saline with 10% DMSO), (b) Group II: control B: (NP), (c) Group III: Low dose BBR-NP (2mg/kg), (d) Group IV: High dose BBRNP(4mg/kg), (e) Group V: Low dose BBR(2mg/kg), (f) Group VI: High dose BBR(4mg/kg).

TUNEL staining showed that renal apoptosis was markedly affected by treatment with either BBR or BBR-NP, both in the 6h and 24h situation (Figure 5). BBR-NP greatly reduced renal apoptosis followed by BBR group. High dose BBR-NP group reduced renal apoptosis better than low group BBR group. The same goes for high dose BBR group and low dose BBR group.

### Potential protection mechanism of BBR-NP on renal I/R injury

**Figure 6:**
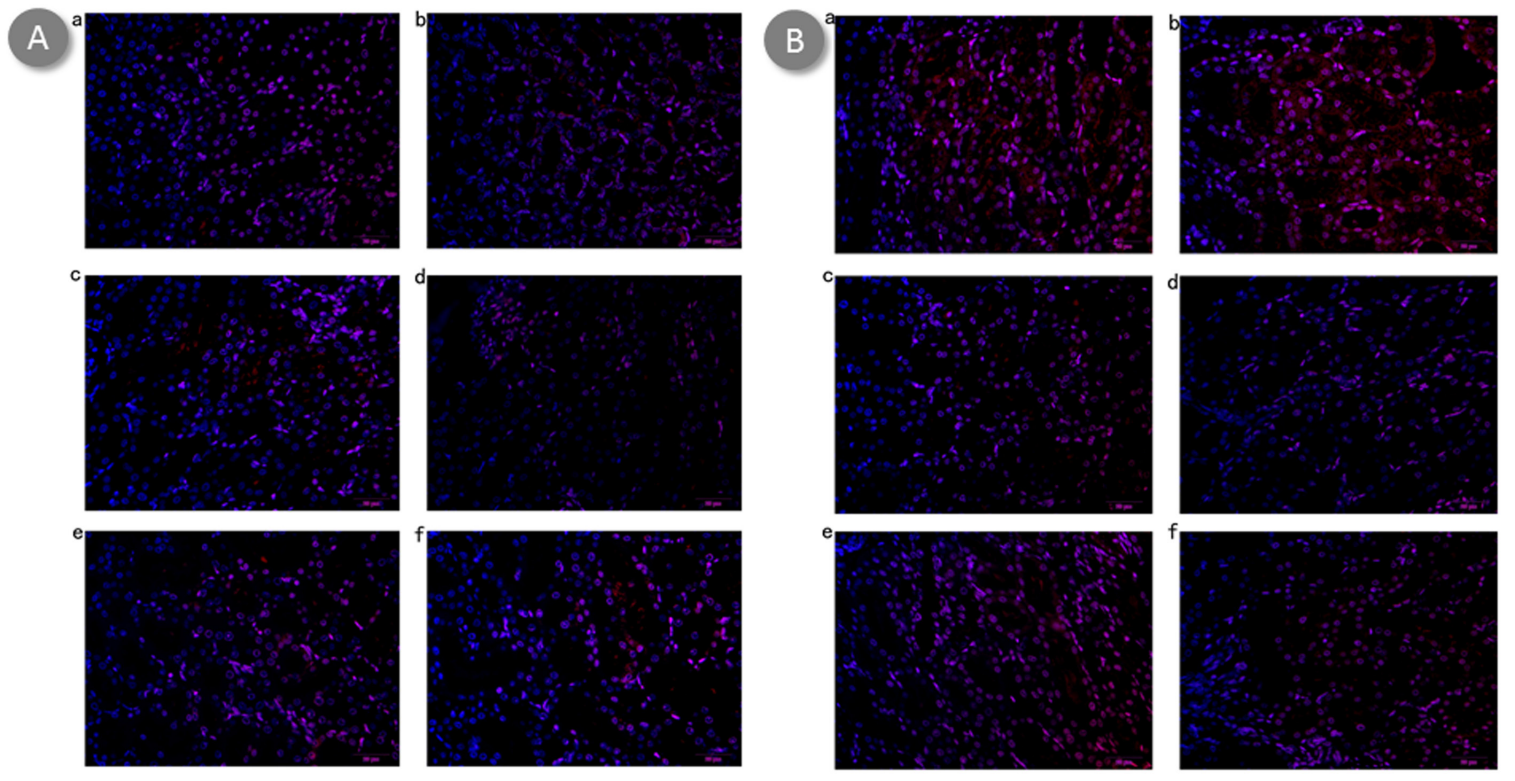
Effect of BBR-NP on renal I/R injury-induced alteration in ROS production using DCFH-DA at 6h (A) and 24h (B) **A**. (a) control A: (saline with 10% DMSO), (b) Group II: control B: (NP), (c) Group III: Low dose BBR-NP (2mg/kg), (d) Group IV: High dose BBRNP(4mg/kg), (e) Group V: Low dose BBR(2mg/kg), (f) Group VI: High dose BBR(4mg/kg). **B**. (a) control A: (saline with 10% DMSO), (b) Group II: control B: (NP), (c) Group III: Low dose BBR-NP (2mg/kg), (d) Group IV: High dose BBRNP(4mg/kg), (e) Group V: Low dose BBR(2mg/kg), (f) Group VI: High dose BBR(4mg/kg).

Overproduction of intracellular ROS is one of the mechanisms involved in renal I/R injury. In our experiment, ROS were detected by DCFH-DA in the kidney with I/R injury. As shown in Figure 6, significantly reduced background fluorescence can be observed low dose BBR-NP group compared with control A and control B group. Even better is the high dose BBR-NP group. Low dose BBR group also showed weak background fluorescence compared with that of control A and control B group, high dose BBR group showed better outcome but still not comparable to the corresponding BBR-NP group.

**Figure 7:**
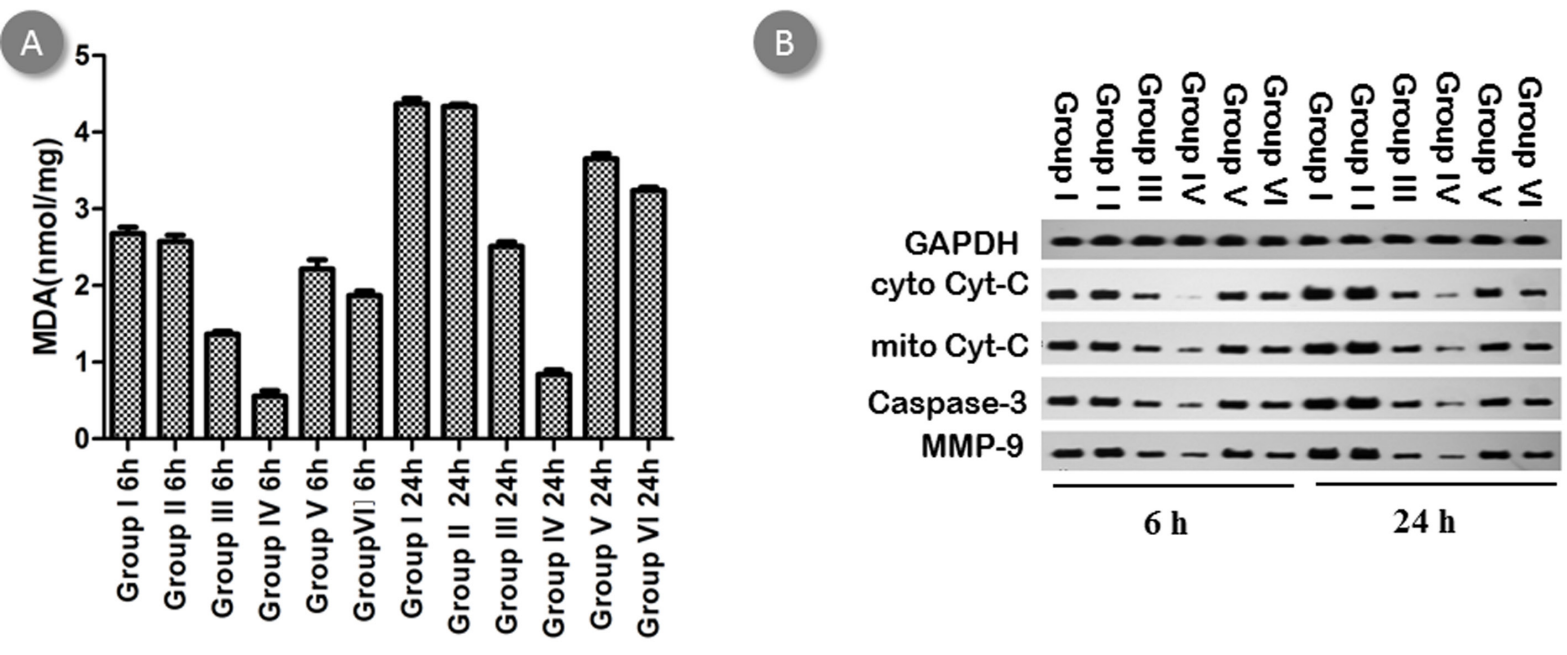
Effect of BBR-NP on renal I/R injury-induced alteration in (A) MDA production at 6h and 24h by TBARS kits and in (B) cytoplasm Cyt-C, mitochondria Cyt-C, Caspase-3 and MMP-9 by western blot Group I: control A (saline with 10% DMSO), Group II: control B: (NP), Group III: Low dose BBR-NP (2mg/kg), Group IV: High dose BBRNP(4mg/kg), Group V: Low dose BBR(2mg/kg), Group VI: High dose BBR(4mg/kg).

Oxidant stress was evaluated by measuring tissue MDA, which was detected by TBARS kits according to the manufacture's instruction. There was significant and dose-dependent degradation in renal MDA level upon treatment with BBR and BBR-NP. In addition, renal MDA levels were drastically degraded in BBR-NP group as compared to BBR group (Figure 7A).

Hydroxyl free radicals destroy the cell membrane [15, 16], release cytochrome C (Cyt C) and activate apoptosis [17]. ROS can also activate the matrix metalloproteinases 9 (MMP 9) [18, 19], leading to the induction of TNF alpha and pro-inflammatory factor such as IL-1, which also contribute to renal I/R injury. We test cytoplasm Cyt-C, mitochondria Cyt-C, Caspase-3 and MMP-9 by western blot. There was significant and dose-dependent degradation in renal cytoplasm Cyt-C, Caspase-3 and MMP-9 level observed by administration of BBR and BBR-NP. Mitochondria Cyt-C significantly increased in both BBR and BBR-NP group. In addition, renal cytoplasm Cyt-C, Caspase-3 and MMP-9 levels were drastically degraded in BBR-NP group as compared to BBR group. Mitochondria Cyt-C significantly increased in BBR-NP group compared with that in BBR-NP group (Figure 7B).

## DISCUSSION

We investigated the effects of BBR-NP on renal I/R injury. Administration of BBR-NP resulted in protection both morphologically and functionally and is superior to BBR alone. Results showed that treatment with BBR-NP prevented oxidative stress and subsequent apoptosis in renal H/R injury. Mechanisms investigation showed that the protective effects of BBR-NP may be largely attributable to the oxidative stress pathways and mitochondrial stress pathways.

BBR holds multiple effects such as anti-inflammatory [20], antimicrobial [21] and antipyretic activities [22]. BBR has been widely used to treat Diarrhea-Predominant Irritable Bowel Syndrome [23], polycystic ovary syndrome [24] and lung disease in the clinic [25]. Recent studies showed that BBR can protect renal IR injury via caspase-mitochondria-dependent pathway [11]. However, its application in oral administration is limited mainly due to its poor aqueous solubility, low gastro-intestinal absorption and rapid metabolism [26]. A potential solution to these problems is to utilize NP formulations, which have been proved to improve bioavailability and efficacy of loaded water-insoluble drugs [27]. In our study, we investigated the effects of BBR-NP on renal I/R injury. We found that BBR-NP significantly decreased the serum urea nitrogen levels and creatinine levels in renal I/R models. Significantly-reduced damages in BBR-NP group versus control can be observed with both HE staining and transmission electron microscopy. Besides, this effect is dose-dependent and more superior than the corresponding BBR group. All these results demonstrated the protective effect of BBR-NP on renal I/R injury both functionally and morphologically.

It was reported that oxidative stress induced by ROS was one of the most important mechanisms underlying renal I/R injury [28]. Excessive ROS triggers lipid peroxidation in cell membranes and thus causes cell components destruction and cell death [29]. MDA is the end-product of lipid peroxidation and therefore is commonly used for biomarkers of oxidative stress. In our study, significant decrease in ROS, MDA were observed in both high- and low-dose BBR-NP groups compared with that of BBR groups and control groups. Our study demonstrates that BBR-NP treatment significantly decreases oxidative stress as demonstrated by reducing renal MDA levels following renal I/R injury.

Oxidative stress can lead to apoptosis, which plays vital roles in the pathogenesis of renal I/R injury. The anti-apoptotic effect of BBR-NP on renal I/R injury was therefore tested. As showed in TUNNEL staining under inverted contrast microscope, less apoptotic morphological changes were observed in BBR group and BBR-NP group compared with controls.

During mitochondrial stress, the expression levels of Bax and Bcl-2 are changed, Cytochrome C is released from mitochondria and the apoptotic effector protein caspase-3 is activated, resulting in cell apoptosis [30]. The decreased cytoplasm Cyt-C and increased mitochondria Cyt-C indicated that the cells had undergone apoptosis by a mitochondrial-dependent pathway, while the cleavage of Caspase-3 reveals the activation of apoptosis. We next checked the expression of Caspase-3 by western blot, finding that caspase-3 was downregulated in BBR-BP group compared with BBR group or control group.

It has been reported that the over-production of ROS induces MMP-9 expression in kidneys [31]. Therefore, we also tested MMP9 by western blot. Results showed that MMP-9 was downregulated in BBR-NP group versus BBR group and control group, indicating that BBR-NP protected renal I/R through degradation of oxidative stress and mitochondrial stress pathways.

There are some limitations in our study. First of all, the experiment is relevantly small one with only 12 rats included. Secondly, we only test the effect of BBR-NP on renal I/R injury induced apoptotic protein markers, the cross-talk between oxidative stress and mitochondrial stress have not been investigated. What is more, the dosage-dependent effects is not enough as we only included two doses. Further investigations are still needed.

## CONCLUSIONS

This study demonstrated the renal protective effect of BBR-NP and its superior bioavailability. Administration of BBR-NP resulted in protection both morphologically and functionally and is superior to BBR alone. Results showed that treatment with BBR-NP prevented oxidative stress and subsequent apoptosis in renal H/R injury. Mechanisms of the protective effects may be largely due to the degradation of oxidative stress and mitochondrial stress pathways.

## MATERIALS AND METHODS

### Experimental animals and research protocol approval

*In vivo* studies were carried out using 12 male Sprague-Dawley rats (weight, 180-200g). Right nephrectomy was performed and left renal ischemia was induced by left renal artery occlusion for 45 minutes [19]. Upon completion of surgical procedures, the animals were randomly allocated into six groups as follows: Group I: control A: (saline with 10% DMSO), Group II: control B: (NP), Group III: Low dose BBR-NP (2mg/kg), Group IV: High dose BBRNP(4mg/kg), Group V: Low dose BBR(2mg/kg), Group VI: High dose BBR(4mg/kg). All of them were done by tail injection. Each group was then divided into the 6 hour group and the 24 hour group, which means 6 hours or 24 hours after the construction of renal I/R injury model. The animal protocol was reviewed and approved by the Institutional Animal Care and Use Committee of Xuzhou Medical University.

### Conformation of renal BBR-NP enrichment

BBR-NP were prepared by a nanoprecipitation method as previously described with minor modification [32–34]. Fluorescence labeled BBR-NP was injected 6 hours before the renal I/R injury rats were anesthetized, then the kidney were harvested and cut coronally. Fluorescence images of BBR-NP generated in kidney were visualized using fluorescence microscope. The fluorescence concentration of the samples was analyzed to confirm the enrichment of BBR-NP in kidney. Similar dose of BBR-NP or BBR without fluorescence were injected in renal I/R injury rats, plasma BBR was measured at 10 minutes, 30 minutes, 60 minutes, 3 hours, 6 hours, 12 hours and 24 hours by HPLC(High-performance liquid chromatography), BBR concentration in kidney was measure at 6 hours and 12 hours by HPLC.

### Renal function and morphological analysis

Blood was obtained at 6 hours and 24 hours after renal I/R injury for serum urea nitrogen and creatinine in a clinical laboratory. Six and 24 hours after renal I/R injury, the rats were anesthetized, left kidney was isolated and fixed for histopathological evaluation with 4% buffered paraformaldehyde solution and embedded in paraffin. Four micrometer thick paraffin sections were dewaxed and then brought to water through graded ethanol. H & E stained sections were graded for the presence of tubular cell necrosis, glomerular hypertrophy, medullary congestion, cytoplasmic vacuolization and cytoplasmic eosinophilia. Samples were then observed using a scanning electron microscope to elucidate the influence of BBR-NP on scaffold micro-architecture.

### Renal apoptosis measurement

Six and 24 hours after renal I/R injury, the rats were anesthetized, left kidney was isolated and were fixed in 4% paraformaldehyde. The fixed tissues were then embedded in paraffin, and TUNEL staining was performed according to the manufacturer's instructions. All nuclei were stained by DAPI. The apoptotic index (ie, the number of TUNEL-positive nuclei/total number of nuclei counted ×100%) was calculated in a blinded manner.

### Detection of intracellular ROS

Six and 24 hours after renal I/R injury, the rats were anesthetized, left kidney was isolated and and cut coronary. DCHF-DA was used to detect intracellular generation of ROS according the instructions. The obtained values were expressed as folds of the controls. Typical images of intracellular ROS generation were obtained based on the green fluorescence of DCHF. The lighter the fluorescence, the greater the ROS generation.

### Detection of malondialdehyde (MDA)

Levels of MDA in kidney was detected by thiobarbituric acid (TBA) colorimetric method using commercially available kits which were purchased from the Jiangsu Kaiji Bioengineering Institut (KGT003). All procedures were performed according to the manufacturers` recommendation in the kit manuals.

### Western blot analysis

Six and 24 hours after renal I/R injury, the rats were anesthetized, left kidney was harvested. Protein levels of Cyt-C, Caspase-3and MMP-9 were analyzed by Western blot as described previously. [20] Briefly, cell lysates were prepared, electrotransferred, and then immunoblotted with anti-Cyt-C (Abcam, ab133504), anti-Caspase-3 (Abcam, ab44976) and anti-MMP-9 (Abcam, ab38898). Detection was performed with Western blotting reagent ECL (Amersham), and chemiluminescence was exposed by the filters of Kodak X-Omat films. After normalizing the bands with the actin control, data analysis was finished by Image Pro Plus (Media Cybernetics, Silver Spring, MD, USA), measuring the densities of immunoreactive bands.

### Statistical analysis

Data were expressed as the mean ± SD. Statistical analysis was based on student's *t-*test or one-way ANOVA analysis with SPSS 11.5 software (SPSS, USA). The accepted level of significance was *P* < 0.05.
